# High‐protein vegan and omnivorous diets improve peripheral insulin sensitivity to a similar extent in people with type 2 diabetes

**DOI:** 10.1111/dom.16100

**Published:** 2024-11-27

**Authors:** Gráinne Whelehan, Marlou L. Dirks, Sam West, Doaa R. Abdelrahman, Andrew J. Murton, Tim J. A. Finnigan, Benjamin T. Wall, Francis B. Stephens

**Affiliations:** ^1^ Department of Public Health and Sport Sciences, Faculty of Health and Life Sciences University of Exeter Exeter UK; ^2^ Human and Animal Physiology Wageningen University Wageningen The Netherlands; ^3^ Department of Surgery University of Texas Medical Branch Galveston Texas USA; ^4^ Sealy Center of Aging University of Texas Medical Branch Galveston Texas USA; ^5^ New Era Foods Rudby Lea Hutton Rudby UK

**Keywords:** glycaemic control, insulin resistance, insulin secretion, type 2 diabetes

## Abstract

**Background:**

High‐protein diets have been recognized as a potential strategy in the nutritional management of type 2 diabetes (T2D). Mycoprotein is a high‐fibre, high‐protein food ingredient previously shown to improve acute glycaemic control. We determined whether incorporating mycoprotein into a high‐protein vegan diet would improve glycaemic control to a greater extent than an isonitrogenous omnivorous diet in people with T2D.

**Methods:**

Seventeen adults (*f* = 5, age = 58.3 ± 8.3 years, BMI = 32.9 ± 4.7 kg∙m^−2^, HbA1c = 60 ± 15 mmol∙mol^−1^) with T2D were randomly allocated to a 5‐week eucaloric high‐protein (30% energy from protein) diet, either an omnivorous diet (OMNI; 70% protein from omnivorous sources) or an isonitrogenous, mycoprotein‐rich, vegan diet (VEG; 50% protein from mycoprotein). Glycaemic control was assessed using a two‐step hyperinsulinaemic‐euglycaemic clamp (HEC) with D‐[6,6‐^2^H_2_] glucose infusion, a mixed‐meal tolerance test (MMTT) and continuous glucose monitoring.

**Results:**

The rate of glucose disappearance (RdT), glucose disposal rate and endogenous glucose production, as well as postprandial time‐course of blood glucose, serum insulin and C‐peptide were assessed during the HEC and MMTT, respectively. Both groups had improved peripheral insulin sensitivity (intervention effect, *p* = 0.006; increased RdT/Insulin of 1.0 ± 0.6 and 1.0 ± 0.3 mg kg^−1^ min^−1^ in OMNI and VEG, respectively), HbA1c (intervention; *p* = 0.001) and glycaemic variability (intervention; *p* = 0.040; increased time in‐range of 11.8 ± 9.3% and 23.3 ± 12.9% in OMNI and VEG). There were no improvements in hepatic insulin sensitivity or in postprandial blood glucose and serum C‐peptide (*p* > 0.05) during the MMTT.

**Conclusions:**

High‐protein diets, whether predicated on vegan or omnivorous proteins, can improve glycaemic control by increasing peripheral insulin sensitivity in people with T2D.

## 1 INTRODUCTION

Dietary intake is an important modifiable factor in the management of type 2 diabetes (T2D). Dietary guidelines to date have focused on achieving a caloric deficit, as T2D remission has been achieved in a number of individuals following sufficient weight loss.^1^, ^2^ Although there is no consensus on the optimal dietary macronutrient composition for patients with T2D,^3^, ^4^ several studies have observed an improvement in glycaemic control when following a high‐protein diet.^5^, ^6^, ^7^, ^8^ The level of protein normally recommended in a ‘healthy’ diet is determined by the minimum daily intake required to maintain nitrogen balance, typically equating to ~10–15% of total energy intake,^9^ but the benefits on glycaemic control, including lower 24‐h blood glucose and decreased HbA1c, have been demonstrated at 30% of energy intake.^6^, ^8^ However, the mechanisms (i.e. enhanced insulin secretion and/or action) underlying the improvement in glycaemic control following a high‐protein diet remain unclear.

Mycoprotein, a high‐fibre, high‐protein food ingredient, has been implicated in the improvement of acute postprandial glycaemia and/or insulinaemia.^10^, ^11^ This improvement is likely due to the largely insoluble fibre composition of mycoprotein, as insoluble fibre has been shown to improve peripheral insulin sensitivity and postprandial glycaemia in the absence of any effect on glucose absorption.^12^, ^13^ Habitual high dietary intakes of insoluble fibre have also been linked to a reduced risk of developing T2D in observational studies,^14^, ^15^, ^16^ but there are limited randomized controlled trials to explore the mechanisms behind this improvement in glycaemic control (independent of weight loss). Daily mycoprotein consumption lowered blood glucose concentrations after 4 weeks in adults who were obese or overweight,^17^ and a trend towards reduced fasting blood glucose concentrations was observed after 1 week of daily consumption in young healthy adults.^18^ However, the former study observed this improvement in combination with weight loss, making it difficult to discern the effects of mycoprotein consumption independent of the known benefits of weight loss on glycaemic control. The latter study was undertaken in metabolically healthy individuals, and thus any improvements in glycaemic control may have been too subtle to detect in this population. Moreover, in both studies, the measures of glycaemic control used did not distinguish between the multifactorial physiological process by which mycoprotein may affect blood glucose concentrations (i.e. improved insulin secretion and/or action). Thus, it remains to be determined how daily mycoprotein consumption improves glycaemic control in a metabolically compromised population, independent of weight loss.

This study examined whether incorporating mycoprotein into a high‐protein vegan diet could enhance glycaemic control more than an isonitrogenous omnivorous diet in people with T2D. True ‘free‐living’ glycaemic variability was measured using continuous glucose monitoring.^19^ We assessed insulin sensitivity using a hyperinsulinaemic‐euglycaemic clamp combined with D‐[6,6‐^2^H_2_] infusion^20^ and insulin secretion using a mixed‐meal tolerance test.^21^ We hypothesized that a 5‐week high‐protein, mycoprotein‐rich, vegan diet would improve peripheral insulin sensitivity to a greater extent than an isonitrogenous omnivorous diet in people with T2D.

## 2 METHODS

A total of 17 participants completed the present study (Table 1). Thirty participants were initially enrolled, nine were withdrawn or dropped out and four were excluded from analysis as they did not consume the mycoprotein products provided (Figure S1). All glucose‐lowering, lipid‐lowering and antihypertensive medications were stable at least 3 months before taking part in the study and remained unchanged during participation. Eligibility criteria included; aged between 40 and 70 years, BMI between 27 and 45 kg m^−2^, T2D diagnosis and a HbA1c above 43 mmol mol^−1^. Exclusion criteria included; treatment with insulin, sulfonylureas or anti‐obesity drugs, weight loss of >5 kg in the 6 months prior to enrolment, an estimated glomerular filtration rate below 30 mL min^−1^, heart failure, known cancer, myocardial infarction or pregnancy. Data collection was conducted from September 2021 to December 2022. Ethical approval was obtained from the National Health Service Health Research Authority (reference number 21/YH/0102). The trial was registered on clinicaltrials.gov as NCT05615558. This study was conducted in accordance with the Declaration of Helsinki.

**TABLE 1:** Participant characteristics.

	OMNI	SD	VEG	SD	*p* value
Females/males	4/5	–	1/7	–	–
Weight, kg	100.6	26.9	97.7	15.2	0.790
Height, cm	172.1	11.4	173.8	7.2	0.726
BMI, kg m^−2^	33.4	5.6	32.3	3.6	0.618
Age, years	58.7	9.4	57.8	7.3	0.851
eGFR	89.1	3.4	87.7	5.2	0.482
HbA1c, mmol mol^−1^	57.6	14.2	62.4	15.6	0.516
Years since diagnosis	5.6	4.2	4.5	2.5	0.504
FPG, mmol L^−1^	6.9	1.6	7.9	1.6	0.258
FSI, uU L^−1^	24.9	10.4	27.3	13.5	0.688
HOMA‐IR	7.5	3.1	9.8	5.9	0.311

*Note*: Data analysed using independent samples *t* test.

Abbreviations: BMI, body mass index; eGFR, estimated glomerular filtration rate; FPG, fasting plasma glucose; FSI, fasting serum insulin; HbA1c, glycated haemoglobin; HOMA‐IR, homeostatic model of insulin resistance.

Participants had an initial screening phone call to determine basic eligibility (i.e. diagnosed with T2D, BMI and age within acceptable range, and taking appropriate medication). Following the initial phone call, participants attended a screening visit at the University of Exeter Nutritional Physiology Research Unit laboratories. After providing written informed consent, participants were allocated using simple randomization to a high‐protein diet; either an omnivorous diet (OMNI; 70% protein from omnivorous sources) or an isonitrogenous mycoprotein‐rich vegan diet (VEG; 50% protein from mycoprotein).

### 2.1 Experimental overview

The study was designed as a randomized, parallel groups 5‐week dietary intervention. Two weeks before commencing the study, the participants recorded habitual physical activity for 1 week using Gene‐activ watches (Activinsights, Kimbolton, UK), habitual dietary intake using weighed food diaries^22^ and interstitial glucose concentration using continuous glucose monitors (Dexcom G6, Dexcom Inc., Edinburgh, UK). One week before commencing the study, participants underwent a mixed‐meal tolerance test (MMTT) and, at least 3 days after the MMTT, a two‐step hyperinsulinaemic‐euglycaemic clamp (HEC). The participants commenced the 5‐week dietary intervention the day after completing the HEC. During the final week of the dietary intervention, the participants again recorded physical activity and interstitial glucose concentration. The MMTT and HEC were repeated after the intervention.

### 2.2 Dietary intervention

Energy intake requirements were calculated as basal metabolic rate^23^ multiplied by a physical activity factor of 1.6.^24^ Dietary control consisted of a 5‐week provision of all food intake for participants, to ensure that the correct amount of energy and distribution of macronutrients was consumed, that is, a high‐protein diet of 30% energy from protein and 35% energy from carbohydrate and fat. The provision of all food also reduces participant burden by reducing choice, shopping time and cost, which increase the likelihood of adherence.^25^ Target mycoprotein consumption was 210 g per day for participants randomized to VEG.^17^, ^18^ Seven‐day diet plans were compiled with a 3‐day rotation. Daily meal plans consisted of breakfast, lunch, dinner and two snacks (sample daily plan Table S3). All food was ordered from a local supermarket (Tesco, Tesco PLC, UK) to the laboratory and weighed out according to the meal plan. Participants collected their food and the 7‐day meal plan from the laboratory on a weekly basis. An adherence diary was also provided for participants to list any changes made to the diet plan.

### 2.3 Metabolic testing

An MMTT was performed at baseline and post‐intervention to measure postprandial blood glucose, serum insulin and C‐peptide response and, consequently, to calculate insulin secretion rate and β‐cell function. The HEC was conducted to assess insulin sensitivity pre and post the dietary intervention.^20^ Details of the procedures are outlined in the Supplementary Material. Calculations for glucose disposal rate, rate of glucose disappearance, endogenous glucose production, β‐cell function and metabolic clearance rate are described in detail in the Supplementary Material.

### 2.4 Sample analysis

Insulin and C‐peptide concentration were measured in serum by enzyme immunoassay sandwich technique using a commercially available kit (DRG Insulin ELISA, EIA‐2935 and DRG C‐Peptide ELISA EIA‐1293, DRG International Inc., New Jersey, USA). Total‐, low‐density lipoprotein (LDL) and high‐density lipoprotein (HDL) cholesterol and triglycerides (TG) were determined in serum by the Blood Sciences Academic Department at the Royal Devon University Healthcare NHS Foundation Trust. Total and HDL cholesterol and triglycerides were measured using enzymatic colourimetric assays on the Cobas c 702 module (using CHOL2, HDLC3 and TRIGL packs, respectively; Roche Diagnostics). LDL cholesterol was calculated using the Friedewald formula as previously described.^26^

### 2.5 Statistical analyses

The primary outcome was rate of disappearance of glucose during the high‐dose insulin infusion, which reflects peripheral insulin sensitivity. Effect size and sample size were calculated using data from Weickert et al.^27^ that showed *n* = 18 was sufficient to detect a 10% increase in insulin sensitivity (‘M’) during the HEC after 5 weeks on a high cereal fibre diet. Due to dropouts, we did not recruit the target number of participants for this study. However, a post hoc power calculation revealed an *n* = 1716 would have been necessary to achieve a 20% difference between OMNI and VEG in peripheral insulin sensitivity. Therefore, if we had recruited the target number of participants, it would likely not have affected the results of the study. Data are presented as mean ± SD, unless otherwise stated. Data were analysed using three‐way repeated measures ANOVAs. Independent variables for the MMTT analyses are time × group × intervention; time refers to timepoints during the MMTT, and group refers to either OMNI or VEG and intervention refers to pre‐ and post‐intervention timepoints. Independent variables for the HEC analyses are insulin × group × intervention, whereby insulin refers to the state of insulinaemia during the HEC, that is, basal, Step 1 (low‐dose; 30 mU m^2^ min^−1^) and Step 2 (high‐dose; 80 mU m^2^ min^−1^). In the event of a significant main effect, post hoc analysis using Tukey's multiple comparisons tests was performed. Two‐way ANOVAs were used to assess differences between the dietary groups for summary measures (e.g. AUCs). Blood glucose concentration, serum insulin concentration, serum C‐peptide concentration, insulin secretion rate, β‐cell function, 24 h interstitial glucose, energy expenditure and carbohydrate and fat oxidation represent our secondary measures.

## 3 RESULTS

### 3.1 Dietary intervention

Habitual energy intake in OMNI and VEG increased during the dietary intervention (intervention effect; *p* = 0.010) (Table S2). Goldberg's equation to estimate under‐reporters^28^ revealed only two under‐reporters in OMNI (values of 0.8 and 0.9) for habitual dietary intake, but these participants were not excluded from the analysis. During the dietary intervention, VEG consumed 237 ± 62 g of mycoprotein which contributed to an additional 22.2 ± 4.2 g fibre from Quorn products (Figure S2). Physical activity did not change during the intervention from habitual levels (Table S5).

**FIGURE 1:**
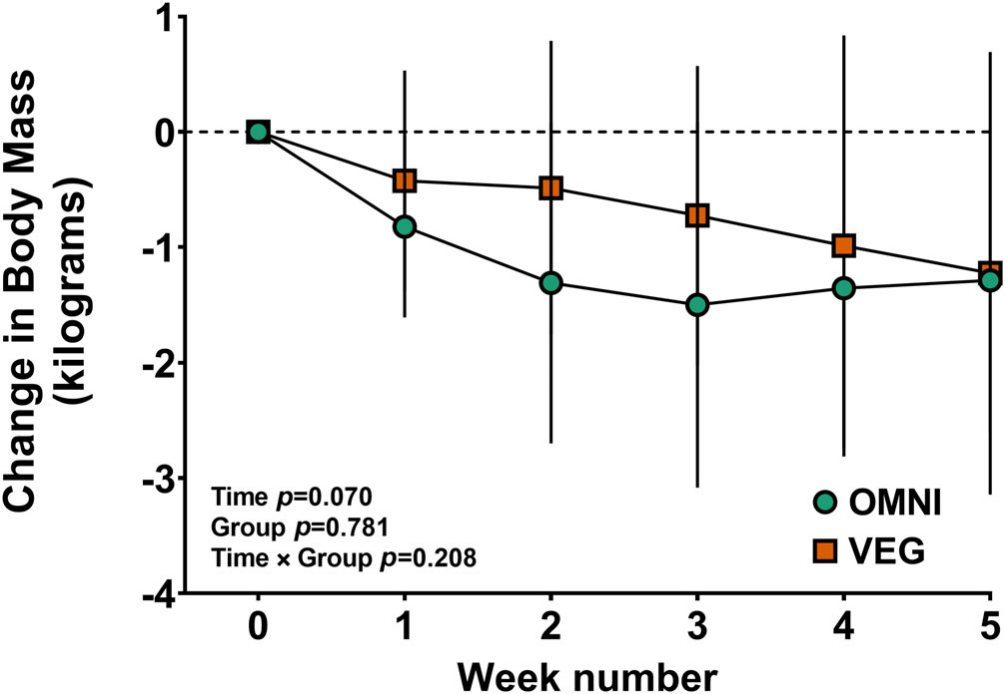
Weekly change in body mass from the week before starting the dietary intervention (week 0). Error bars depict SD. Circles and squares represent the OMNI and VEG group, respectively.

Body mass at baseline was 100.6 ± 26.9 and 98.7 ± 12.9 kg, in OMNI and VEG, respectively. The mean change from week 0 to week 5 was −1.3 (range 0.7 to −3.1) kg and − 1.2 (range 1.2 to −5.0) kg (Figure 1) in OMNI and VEG (time effect; *p* = 0.070), which was not different between groups (time × group effect; *p* = 0.208).

Fasting circulating metabolite concentrations are shown in Table 2. Fasting blood glucose, serum insulin and serum C‐peptide concentration all trended towards a group × intervention interaction, largely due to no change in fasting concentrations in VEG, and a trend towards increased blood glucose (6.8 ± 1.8 to 7.4 ± 2.6 mmol‧L^−1^) and serum insulin (173 ± 73 to 192 ± 88 pmol‧L^−1^), and decreased serum C‐peptide (1906 ± 310 to 1691 ± 282 pmol‧L^−1^) concentration, in OMNI. The intervention decreased HbA1c in both groups (intervention effect; *p =* 0.001).

**TABLE 2:** Fasting metabolite concentrations.

	OMNI		VEG		*p‐values*		
	Pre	Post	Pre	Post	*Group*	*Intervention*	*Group × intervention*
Blood glucose, mmol‧L^−1^	6.8 ± 1.8	7.4 ± 2.6	7.3 ± 1.5	7.3 ± 1.6	0.800	0.430	0.058
Serum insulin, pmol‧L^−1^	173 ± 73	192 ± 88	186 ± 88	172 ± 73	0.913	0.189	0.086
Serum insulin, mU‧mL^−1^	24.9 ± 3.5	30.3 ± 5.2	27.3 ± 4.8	26.5 ± 4.1	0.913	0.189	0.086
Serum C‐peptide, pmol‧L^−1^	1906 ± 930	1691 ± 847	1855 ± 821	1934 ± 908	0.730	0.974	0.080
HbA1c, mmol‧mol^−1^	59 ± 5	53 ± 6	62 ± 6	53 ± 4	0.677	0.001*	0.292
Plasma lactate, mmol‧L^−1^	1.0 ± 0.1	0.9 ± 0.1	1.1 ± 0.1	0.8 ± 0.1	0.675	<0.001**	0.003*
Total cholesterol, mmol‧L^−1^	4.3 ± 1.0	3.9 ± 1.2	4.1 ± 1.0	3.2 ± 0.9	0.331	<0.001**	0.056
HDL cholesterol, mmol‧L^−1^	1.1 ± 0.3	0.9 ± 0.2	1.0 ± 0.2	0.9 ± 0.2	0.921	<0.001**	0.229
LDL cholesterol, mmol‧L^−1^	2.6 ± 0.9	2.4 ± 1.1	2.3 ± 0.9	1.7 ± 0.7	0.244	0.009*	0.105
Triglycerides, mmol‧L^−1^	1.6 ± 0.5	1.3 ± 0.5	1.7 ± 1.1	1.2 ± 0.6	0.870	0.006*	0.302

*Note*. Data presented as mean ± SD. Variables measured using two‐way ANOVA. Total, HDL, and LDL cholesterol and triglycerides were measured in serum. *p <0.01, **p < 0.001.

### 3.2 Continuous glucose monitoring

Glycaemic variability was reduced post‐intervention in both OMNI and VEG, as percentage time in range (4–10 mmol∙L^−1^) increased from 60.2 ± 39.0% and 46.5 ± 39.9% in OMNI and VEG, respectively, to 72.1 ± 36.5% and 69.9 ± 30.2% (intervention effect; *p* = 0.040) (Figure S3). Mean interstitial glucose concentrations did not change from baseline to post‐intervention in either dietary group (intervention effect; *p* = 0.116).

### 3.3 Mixed‐meal tolerance test

**FIGURE 2:**
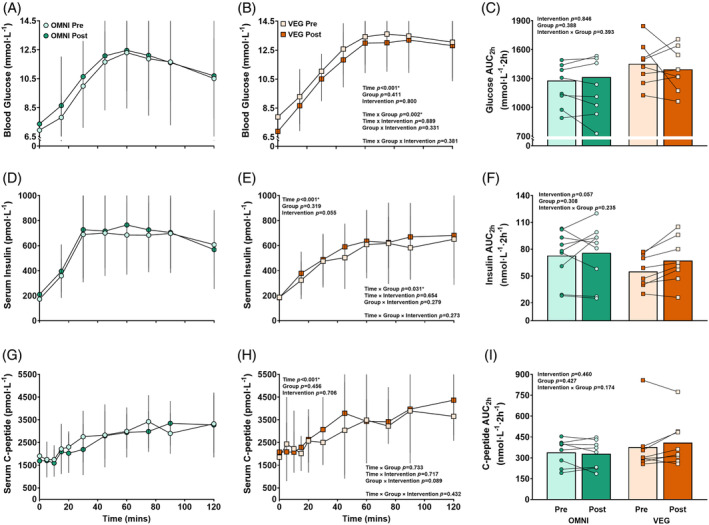
Blood glucose (A, B), serum insulin (D, E) and serum C‐peptide (G, H) concentrations and AUC (C, F, I), respectively) in response to a mixed‐meal tolerance test pre (A, D, G) and post (B, E, H) dietary intervention. Circles represent the response in the OMNI group, and squares represent the VEG group. Lighter shading represents the response to the MMTT pre‐intervention and darker shading represents the response post‐intervention. Time‐course data are analysed by three‐way ANOVA incorporating time‐course (time), dietary group (group) and intervention as independent variables. AUC data are analysed by two‐way ANOVA for pre‐ and post‐intervention values for the OMNI and the VEG dietary groups. Error bars depict SD. *p*‐values are displayed on each graph. Asterisk represents statistical significance *p* < 0.05.

After ingestion of the MMTT, blood glucose, serum insulin and serum C‐peptide concentrations increased (Figure 2A–H) (time effect; *p* < 0.001), but there was no main effect of the intervention or dietary group (*p* > 0.05). The glycaemic and insulinaemic response differed over time between groups (glucose time × group; *p =* 0.002, insulin time × group; *p =* 0.031), due to the different drinks ingested by both groups, causing a greater serum insulin concentration at 30 min in OMNI compared with VEG, both pre‐ and post‐intervention (*p* = 0.012). Glucose AUC_2h_ remained unchanged after the intervention (intervention effect; *p* = 0.846), while insulin AUC_2h_ values trended towards an increase post‐intervention (intervention effect; *p* = 0.057). Insulin secretion rates increased after the ingestion of the MMTT (time effect; *p* < 0.001) (Figure S4A,B), but there was no effect of the intervention (*p* = 0.181) or dietary group (*p* = 0.953). β‐cell function trended towards an increase after the intervention in both groups (intervention effect; *p* = 0.058) (Figure S4C).

### 3.4 Hyperinsulinaemic‐euglycaemic clamp

Mean glucose and insulin infusion rates, as well as blood glucose and serum insulin concentration are displayed in Figure S5A–D.

After initiating the low‐dose insulin infusion (Step 1), blood glucose concentration was allowed to drop to 5 mmol L^−1^. One participant in OMNI and one participant in VEG did not reach a blood glucose concentration of 5 mmol L^−1^ during Step 1 pre‐intervention, and only the participant in VEG did not reach 5 mmol L^−1^ during Step 1 post‐intervention.

Serum insulin concentration increased during the HEC from basal to Step 1 and again from Step 1 to Step 2 (insulin effect; *p* < 0.001) (Figure S6A). A main intervention effect (*p* = 0.012) on serum insulin concentration during the HEC was driven by a lower concentration during Step 2 post‐intervention (insulin × intervention; *p =* 0.044), and post hoc analysis revealed that this occurred in VEG (*p* = 0.017) and not OMNI (*p* = 0.403). A main effect of insulin corresponded with a decrease in serum C‐peptide concentration during the HEC (Figure S6B). There was no main intervention effect (*p* = 0.230), but a group × intervention interaction (*p* = 0.026) with post hoc analysis revealed lower C‐peptide concentration in VEG post‐intervention (*p =* 0.022) and not in OMNI (*p* = 0.667).

**FIGURE 3:**
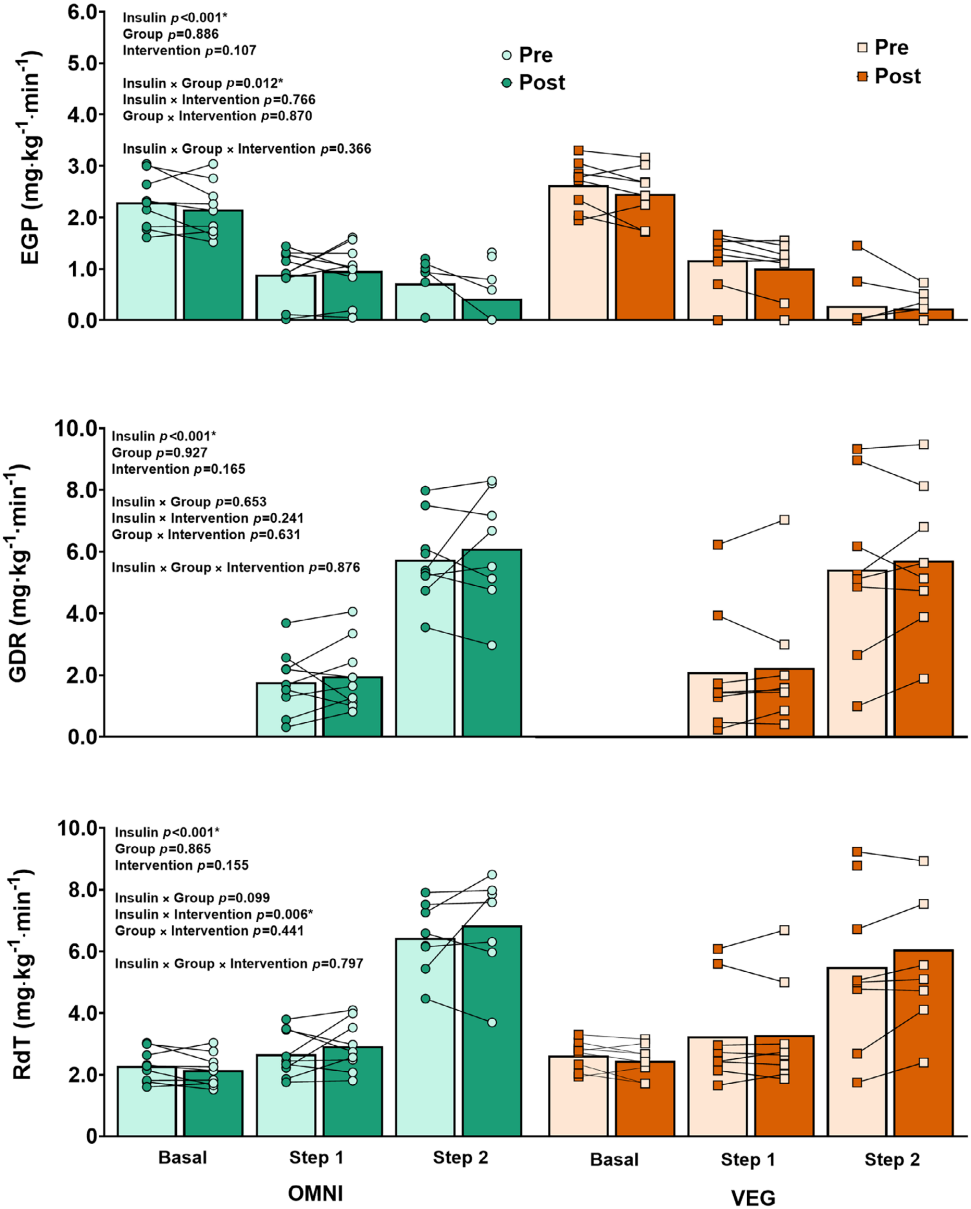
Glucose metabolism during hyperinsulinaemic‐euglycaemic clamOMNI group data shown on the left using circles for individual data and VEG group data shown on the right using squares for individual data. Data analysed using three‐way ANOVA to determine the main and interaction effects of insulin dose (insulin), OMNI or VEG dietary group (group) and intervention (pre and post) on endogenous glucose production (EGP) (A), glucose disposal rate (GDR) (B) and total rate of disappearance of glucose (RdT) (C). *p*‐values are displayed on each graph. Asterisk represents statistical significance *p* < 0.05.

Endogenous glucose production (EGP) decreased during the HEC (insulin effect; *p* < 0.001) (Figure 3A). The percentage suppression of EGP during Step 1, that is, hepatic insulin sensitivity, remained unchanged after the intervention (intervention effect; *p* = 0.107). There was no main group effect (*p* = 0.886), but an insulin × group interaction occurred (*p* = 0.012), due to a greater suppression of EGP during Step 2 of the HEC in VEG (87 ± 19%) compared with OMNI (68 ± 29%). The calculated metrics of insulin resistance are displayed in Table S4.

Both glucose disposal rate (GDR) and total rate of disappearance of glucose (RdT) increased during the HEC (insulin effect; *p <* 0.001) (Figure 3B,C). There was no main intervention or group effect. Both GDR and RdT during Step 2, corrected for steady‐state serum insulin concentration (GDR/insulin and RdT/insulin), that is, whole‐body and peripheral insulin sensitivity, respectively, increased post‐intervention (intervention effect; *p* = 0.014 and *p* = 0.006) (Table S4).

Both carbohydrate oxidation and storage increased during the HEC (insulin effect; *p* < 0.001) (Figure S7A,B), but did not change after the intervention (intervention effect; *p* > 0.05). A trend towards a main group effect for carbohydrate oxidation occurred (*p =* 0.071).

## 4 DISCUSSION

The main finding of the present study was that, in contrast to our hypothesis, eucaloric, isonitrogenous high‐protein vegan (VEG) or omnivorous (OMNI) diets improve peripheral insulin sensitivity (IS) in people with T2D to a similar degree, despite an additional 22 g fibre per day from the mycoprotein‐based products in VEG. Glycaemic control was improved, when measured as decreased HbA1c and free‐living 24‐h glycaemic variability, after a fully controlled high‐protein diet, likely through a 37% increase in peripheral (IS) and 45% improvement in β‐cell function. The current study lends further support for the use of high‐protein diets to lower blood glucose in individuals with impaired glycaemic control.

The improvements in ‘free‐living’ glycaemic variability (17% greater time in range of 4–10 mmol∙L^−1^ glucose), following both of the 30% high‐protein diets and the 8 mmol∙mol^−1^ decrease in HbA1c, corroborate previous findings of 6^6^ and 9^7^ mmol∙mol^−1^ decrease in HbA1c after a similar 5‐week 30% high‐protein diet in people with T2D. However, these previous studies do not provide any mechanistic information on the improvement in glycaemic control. We used a gold‐standard two‐step hyperinsulinaemic‐euglycaemic clamp (HEC) in combination with stable isotope glucose tracers to distinguish between insulin sensitive tissues that produce (e.g. liver) and utilize (e.g. skeletal muscle) glucose. In line with our hypothesis, peripheral IS, as assessed by the rate of disappearance of glucose for a given serum insulin concentration (RdT/I), increased by 37% after both dietary interventions. A previous study showed a comparable increase in whole‐body IS after a 6‐week eucaloric 30% high‐protein diet (both animal and vegan sources), measured as a 12% increase in glucose disposal rate during a 40‐mU·m^−2^ ·min^−1^ HEC, in a population with liver steatosis.^29^ However, the single‐step, low insulin infusion rate HEC did not allow for distinction between IS of glucose production and utilization to be explored. It is interesting to note that we did not observe an increase in hepatic IS, as assessed by the percentage suppression of endogenous glucose production (EGP) during the low‐dose (30‐mU·m^−2^ ·min^−1^) insulin infusion of the HEC, nor a reduction in blood glucose concentration during the MMTT, following either high‐protein diet intervention. This is in contrast to a previous study that has shown an increase in hepatic IS during a low‐dose 12‐mU·m^−2^ ·min^−1^ HEC in individuals with obesity following a 4 day 30% high‐protein diet,^30^ which was associated with a reduction in liver triacylglycerols measured by magnetic resonance. Indeed, the 5‐week 30% high‐protein diet provided by Skytte et al.^6^ where a reduction in fasting plasma glucose was observed in individuals with T2D, also demonstrated a reduction in liver fat. The present study did not measure liver fat, but did demonstrate an improved lipid profile and a reduction in serum triglycerides, possibly reflecting reduced liver lipid delivery and/or production, which was reflected by a trend for a 7% reduction in basal EGP, that is, a beneficial effect at low insulin concentration. Clearly, more research is needed to determine the effect of high‐protein diets on liver lipid content and EGP, but, taken together, it would appear that the major effect of short‐term high‐protein diets on glycaemic control in the present study is at the level of insulin action on peripheral glucose utilization, particularly given that carbohydrate oxidation and storage were greater for a given circulating insulin concentration.

In addition to IS as assessed by the HEC, we also measured β‐cell function to comprehensively discern where the mechanisms of improvement in glycaemic control occurred. β‐cell function, measured as the change in insulin secretion rate over 2 h relative to the change in blood glucose and divided by insulin sensitivity,^31^ trended towards a 45% increase after the intervention. This finding is supported by the observation of decreased circulating insulin concentration and secretion rate during the HEC post‐intervention following both diets (although this was also partly due to increased insulin metabolic clearance rate). There are two potential reasons for this trend. Firstly, the increased dietary protein intake may have caused increased amino acid‐stimulated insulin secretion, as this mechanism of insulin secretion has previously been shown to remain intact in people with T2D.^32^ Secondly, our high‐protein diet necessitated a moderate carbohydrate restriction (35% energy from carbohydrate) over the 5 weeks that may have enabled the β‐cells to ‘rest’, as demonstrated by the 17% increased time in‐range, possibly representing the relationship between reduced exogenous carbohydrate and systemic glucose concentration. Previous research has demonstrated an improvement in β‐cell function following somatostatin or diazoxide administration, or aggressive insulin treatment, designed to allow the β‐cells to rest.^33^, ^34^, ^35^ Skytte et al. also observed a 31% improvement in β‐cell function during a mixed‐meal after a high‐protein diet, suggesting this was due to less ‘stress’ on the β‐cells from the carbohydrate‐restricted diet (30% total energy).^5^ Regardless of the mechanism, the importance of β‐cell function in the pathophysiology of T2D is widely acknowledged,^36^ with the return of β‐cell function identified as one of the key drivers of sustained T2D remission.^37^, ^38^

It is difficult to elucidate why we did not observe a difference in IS or β‐cell function between the high‐protein VEG and OMNI diets. As with all dietary intervention studies that manipulate macronutrient composition, the increase in dietary protein results in a reduction in dietary carbohydrate, making it difficult to discern whether the improvements in glycaemic control were due to protein per se. Moreover, the unique composition of mycoprotein (6% fibre from soluble β‐glucan and insoluble chitin), and the nature of trying to match the VEG and OMNI diets for similar meals, resulted in both groups consuming a high‐fibre diet, which is known to affect glycaemic control.^39^ We provided over 40 g fibre per day during the 5‐week dietary intervention, surpassing current recommended daily intakes of >35 g for people with diabetes^39^, ^40^ and also exceeding the habitual intakes of the participants (around 25 g/day). Indeed, Robertson et al. demonstrated a 12% increase in IS during a 35‐mU·m^−2^ ·min^−1^ HEC following 4 weeks of supplementation with 30 g per day resistant starch in young healthy adults, possibly due to the increase in short‐chain fatty acid availability, which is known to inhibit adipose tissue lipolysis.^41^ Moreover, Weickert et al. demonstrated a 10% increase in peripheral IS during a 40‐mU·m^−2^ ·min^−1^ HEC following a 6‐week high protein (30%) and high insoluble cereal fibre (>50 g total daily fibre) diet in individuals who were overweight and obese.^27^ Interestingly, the same study demonstrated a 10% decrease in IS following the high‐protein diet with low fibre content (~20 g fibre per day), suggesting that the sub‐optimal amount of dietary fibre may have caused a reduction in IS and that the high‐protein diet per se may not have been beneficial for IS. Indeed, the previously mentioned high‐protein diet studies that observed an improvement in glycaemic control provided a minimum of 26^7^ and 30 g^6^ fibre per day. This would fit with the previously reported dose–response improvement in glucose tolerance observed up to 25 g fibre per day, beyond which a plateau occurs,^42^ and the similar improvement in IS in both the OMNI and VEG diets in the present study, despite a significantly greater amount of fibre in the VEG group (an increase from habitual intakes by 39 to 65 g vs. an increase of 18 g in OMNI to a total of 43 g fibre per day).

A major limitation of the present study was the unblinded design and lack of a ‘normal’ protein control group. Thus, we cannot exclude the fact that any improvement in glycaemic control observed was due to taking part in a controlled dietary study per se. Nevertheless, given that body mass did not change over the 5‐week dietary intervention, a major strength compared to other long‐term dietary experiments, we can be confident that the observations were due to a very significant change in habitual dietary composition. Indeed, the control of body weight, high adherence due to full meal provision, comprehensive physiological measures that allowed the distinction between hepatic and peripheral IS and close monitoring of study participants ensured that we could explore the impact of the OMNI and VEG diet on glycaemic control independent of the insulin‐sensitizing effects of weight loss. Nevertheless, not all of our measures gave the same outcome, in particular a lack of effect of dietary intervention on circulating glucose and insulin during the MMTT. This finding is difficult to reconcile, particularly given the importance of the postprandial period to overall glycaemic control. We chose to use the same MMTT before and after the dietary intervention, whereas previous studies have used a MMTT representing the macronutrient composition of the administered dietary intervention.^5^ Thus, given that a low‐carbohydrate diet can cause a degree of glucose intolerance,^43^, ^44^ it may be that providing the same MMTT post‐intervention represented a relatively larger carbohydrate load, and that interpretation of MMTT (and indeed OGTT) in dietary intervention studies requires more critical insight. For example, we observed a lower serum insulin concentration during the post intervention HEC in the present study, which clearly suggests that the diet altered the inherent metabolism of the participants (e.g. insulin clearance rate), but was not detected within the MMTT, and highlighting again the importance of using the gold‐standard measures in the present study.

To conclude, improvements in glycaemic control, including β‐cell function, peripheral insulin sensitivity, glycaemic variability and HbA1c, following a 30% high‐protein diet, were similar between vegan and omnivorous diets, despite the vegan diet containing 22 g more fibre due to the addition of mycoprotein products. These data confirm that high‐protein and fibre diets are beneficial for improving glycaemic control in people with T2D, primarily by increasing insulin sensitivity. These data also inform future work on the mechanisms behind the role of high‐protein diets in the management of glycaemic control in people with T2D.

## AUTHOR CONTRIBUTIONS

Gráinne Whelehan, Francis B. Stephens, and Benjamin T. Wall designed the research; Gráinne Whelehan conducted the research; Gráinne Whelehan, Andrew J. Murton, and Doaa R. Abdelrahman performed the biological analysis; Gráinne Whelehan and Francis B. Stephens analysed the data and wrote the manuscript; Francis B. Stephens had primary responsibility for the final content; and all authors read and approved the final content. Tim JA. Finnigan was an employee of Marlow Foods; Benjamin T. Wall and Francis B. Stephens are employees of the University of Exeter.

## FUNDING INFORMATION

The project was sponsored by Marlow Foods Ltd. (FBS as grant holder). A.J.M. and D.R.A. are supported in part by a grant from the National Institute of Aging (P30‐AG024832). G.W. and S.W. were supported from a studentship grant in collaboration with Marlow Foods.

## CONFLICT OF INTEREST STATEMENT

The authors declare no conflicts of interest.

### PEER REVIEW

The peer review history for this article is available at https://www.webofscience.com/api/gateway/wos/peer‐review/10.1111/dom.16100.

## DISCLAIMER

The University of Exeter (F.B.S.) was responsible for the study design, data collection and analysis, decision to publish, and preparation of the manuscript. The private partners have contributed to the project through regular discussion.

## Supporting information


Data S1.

## Data Availability

Data are available upon reasonable request from FBS, f.b.stephens@exeter.ac.uk.
