# Supplementary material for: High‐protein vegan and omnivorous diets improve peripheral insulin sensitivity to a similar extent in people with type 2 diabetes
Source: Diabetes Obes Metab. 2024 Nov 27;27(3):1143–52. doi: 10.1111/dom.16100 (PMC11802395; doi:10.1111/dom.16100)
Supplement: Supplementary file 1 — Data S1. [file DOM-27-1143-s001.docx]

High-protein vegan and omnivorous diets improve peripheral insulin sensitivity to a similar extent in people with type 2 diabetes.

*Gráinne Whelehan, Marlou L. Dirks, Sam West, Doaa R. Abdelrahman, Andrew J. Murton, Tim JA. Finnigan, Benjamin T. Wall, Francis B. Stephens.*

**Supplementary Material**

**Methods**

*Continuous Glucose Monitoring*

Continuous glucose monitoring (CGM) was used to measure interstitial glucose concentrations over 7 days at baseline, i.e., habitual glycaemic control pre-intervention, and for the final 7 days of the dietary intervention. The CGM system (Dexcom G4 Platinum) measured glucose concentration in the interstitial fluid in the range of 2-22 mmol∙L^-1^ every 5 min. The CGM sensor was inserted under the skin of the abdomen by a trained researcher and was held in place by an adhesive patch. The receiver of the CGM was ‘blinded’ so that participants did not receive any information on glucose levels while wearing the device.

*Mixed-Meal Tolerance Test*

A mixed-meal tolerance test (MMTT) was performed at baseline and post-intervention in all participants to measure postprandial blood glucose, serum insulin and C-peptide response and, consequently, to calculate insulin secretion and β-cell function. The OMNI group received a milk-based drink and the VEG group received a mycoprotein-based drink. Both drinks were matched for macronutrients (*Supplementary Material Table S1*), including 74 g of carbohydrate (dextrose from MyProtein™, THG plc, Manchester, UK). The high fibre content (1/3 chitin, 2/3 insoluble β-glucan) of mycoprotein provided an additional 10 g fibre to the VEG drink. The OMNI drink contained 250 mL full-fat milk, 50 g dextrose (MyProtein™, THG plc, Manchester, UK), 18.7 g dried skim milk (Tesco Instant Dried Skimmed Milk, Exeter, UK) and 9 g full fat dried milk (NIDO Milk Powder, Nestle, Vevey, Switzerland). The mycoprotein-based VEG drink contained 70 g dextrose, 40 g mycoprotein (freeze-dried mycoprotein was produced and provided by Marlow Foods Ltd, Quorn Foods) and 7 g olive oil (Tesco, Exeter, UK). The participants refrained from alcohol consumption and vigorous activity 24 h before each MMTT and were instructed to use the same mode of transportation to the laboratory on each trial visit. Participants were provided with a ‘ready meal’ (consisting of 50 % energy from carbohydrate, 35 % from fat and 15 % from protein) the evening before each MMTT to consume at 8 pm and were instructed not to eat anything after this meal, in accordance with procedures for standardising postprandial glucose tolerance testing [1]. All participants fasted overnight but could drink water *ad libitum*.

The participants arrived at the laboratory at 08:00. There they rested in a semi-supine position and a cannula was inserted in a retrograde fashion into a dorsal hand vein of the non-dominant arm. The hand was then placed in a heated hand-box (air temperature 55 ºC) to obtain arterialised venous blood samples. A saline drip was attached to keep the cannula patent for repeated blood sampling. At *t*= -10, -5- and -1 min blood samples were taken to measure fasting concentrations of glucose, insulin and C-peptide. At *t*= 0 the test-drink was provided and consumed within 2 min. Blood samples were taken at *t*= 5-, 10-, 15-, 20-, 30-, 45-, 60-, 75-, 90- and 120-min post drink ingestion. Except for the measurement of blood glucose, which was performed immediately, whole blood was dispensed into lithium-heparin and serum-separating tubes (BD vacutainers LH and SST II, BD Diagnostics, Nu-Care, Bedfordshire, UK), which were mixed by inversion (eight to ten times). Lithium-heparin tubes were then centrifuged (4ºC and 3500 *g*) for 10 min to obtain plasma. Serum tubes were left to stand at room temperature for 30 min before centrifugation using the same centrifugation settings. The serum and plasma were then dispensed into 1 mL aliquots and immediately frozen at –20 ºC. At the end of each test-day samples were transferred to storage at -80 ºC until assay.

*Hyperinsulinaemic-euglycaemic clamp*

The hyperinsulinaemic-euglycaemic clamp (HEC) was conducted to assess insulin sensitivity pre and post the dietary intervention [2] and was conducted at least 3 days after the MMTT. The participants arrived at the laboratory at 08:00 and lay semi-supine. As with the MMTT, a retrograde cannula for blood sampling was inserted into a superficial vein on the dorsal surface of the non-dominant hand and placed inside a heated hand-box. A saline drip was attached to keep the cannula patent for repeated blood sampling. A second cannula was inserted into the antecubital vein of the contralateral arm. Thereafter, a primed (4 mg٠kg^-1^ over 3 min), continuous (0.04 mg٠kg^-1^٠min^-1^) infusion of [6,6-^2^H_2_] glucose was administered at *t*=-120 min. At *t*=0 a two-step HEC was performed, with insulin (human Actrapid 100 IU∙mL^-1^, Novo Nordisk, West Sussex, UK) infused into the non-dominant arm at a rate of 30 mU٠m^-2^٠min^-1^ (Step 1; low-dose) for 2.5 h to determine EGP, and then 80 mU٠m^-2^٠min^-1^ (Step 2; high-dose) for a further 2.5 h to determine total rate of glucose disappearance (Rd).

Arterialised-venous blood glucose concentration was measured every 5 min (YSI 2300; Yellow Springs Inc., Ohio, USA) and maintained at 5 mmol٠L^-1^ by a variable rate infusion of 20% glucose spiked with 1% of [6,6-^2^H_2_] glucose. At *t*= 5 h the insulin was stopped, and the glucose infusion was continued for approximately 1 h to stabilise blood glucose before removing the glucose infusion line. During this time subjects were provided a standard lunch meal which included a whole-wheat sandwich, fruit, orange juice and tea or coffee.

Arterialised-venous hand blood samples (8 mL) were taken at baseline and every 15 min during the final 30 min of each step of the HEC for the measurement of plasma [6,6-^2^H_2_] glucose, insulin, and C-peptide concentrations. Blood samples were collected and stored in the same manner as during the MMTT.

Gas exchange measurements for volume of oxygen consumption (VO_2_) and volume of carbon dioxide production (VCO_2_) were taken during the final 30 min of each step of the HEC to calculate oxidative and non-oxidative glucose disposal by standard equations. Rates of whole-body fat and carbohydrate utilisation were calculated using the equations of Frayn [3]

*Calculations*

Glucose disposal rate (GDR) was calculated according to equations by Defronzo *et al* [2]. GDR was calculated relative to body weight at each 5 min time-point and averaged over 15 min intervals. An average of GDR over the final 30 min of the HEC during step 1 and step 2 is reported.

GDR = $\frac{G_{inf}-(\Delta G*\left( BW*0.19 \right))}{5}$

Where *G_inf_* is equal to the amount of glucose infused (i.e., glucose infusion rate converted from ml per hour to mg per 5 min). *∆G* refers to the change in glucose concentration and *0.19 x BW* (body weight in kg) is the glucose space in litres.

RdT was calculated using standard Steele equations for steady-state [4] as tracer infusion rates relative to tracer enrichment.

RdT = $\frac{F_{infusion}}{{IE}_{\left[ 6,6-^{2}H_{2} \right]-glucose}}$

$F_{infusion}$ is the total glucose infusion rate (0.04 mg∙kg^1^∙min^-1^ + 0.01*GDR to account for 10% enrichment of dextrose infusion) and ${IE}_{[6,6-^{2}H_{2}]-glucose}$ are the glucose isotopic enrichments.

EGP was calculated as the difference between RdT and GDR, i.e., $EGP=RdT-GDR$.

Numerous metrics for hepatic and peripheral insulin sensitivity (IS) can be used. In the present study we defined hepatic IS as the percentage suppression of EGP from basal to Step 1 of the HEC (low-dose insulin infusion), i.e., the greater the percentage suppression of EGP the greater the degree of hepatic IS. We also report on a metric of hepatic insulin resistance, defined by the product of basal EGP and fasting serum insulin concentrations, i.e., the higher the value the greater the degree of hepatic IR. We used GDR during Step 2, the high-dose insulin infusion, factored by peripheral insulin concentrations, as an index of whole-body IS, and RdT during Step 2, factored by insulin concentration, as peripheral insulin sensitivity:

Hepatic IS = $\frac{{EGP}_{basal}-{EGP}_{step 1}}{{EGP}_{basal}} x 100$

Whole-Body IS = $\frac{{GDR}_{step2}}{{Insulin}_{step 2}-{Insulin}_{basal}}x 100$

Peripheral IS = $\frac{{RdT}_{step2}}{{Insulin}_{step 2}-{Insulin}_{basal}}x 100$

To calculate insulin secretion rates (ISR) during the MMTT we used a deconvolution method of C-peptide concentrations with a two-pool model, as initially proposed by Eaton *et al* [5], using the population constants proposed by Van Cauter *et al* [6]. Values for C(*t*) were obtained by multiplying the experimentally observed C-peptide serum concentrations by the volume of distribution proposed by Van Cauter [6], and fitting a cubic spline and interpolating at 1 min intervals. The smoothed data was differentiated and integrated in Graphpad Prism and these values were used in the Eaton equation to obtain ISR at 1-min intervals.

As an index of β-cell function we used the slope of the line of the integral of ISR and blood glucose during the MMTT at *t*= 120, factored by peripheral IR. Peripheral IR was calculated as the inverse of peripheral IS as calculated above. This method was based on that used by Gastaldelli and Defronzo [7].

β-cell function = $\frac{\Delta ISR}{\Delta G}\div\frac{1}{{RdT}_{SSPIstep2}}$

To calculate metabolic clearance rate (MCR) during the HEGC we used the method proposed by Gastaldelli [7]. Insulin infusion rates are divided by the steady-state plasma insulin (SSPI) minus the endogenous insulin during the clamp, which is calculated from serum C-peptide concentrations.

MCR = $\frac{{Insulin}_{infusion}}{SSPI-(FPI x \frac{{C-peptide}_{clamp}}{{C-peptide}_{basal}})}$

Gas exchange measurements for volume of oxygen consumption (VO_2_) and volume of carbon dioxide production (VCO_2_) were taken during the same 30 min to calculate oxidative and non-oxidative glucose disposal by standard equations. Rates of whole-body fat and carbohydrate utilisation were calculated using the equations of Frayn [3].

| **CHO oxidation (g·min^-1^):** $\left( 4.55* {VCO}_{2} \right)-\left( 3.21* {VO}_{2} \right)-(0.459 {PRO}_{oxidation})$ |
| --- |
| **FAT oxidation (g·min^-1^):** $\left( 1.67* {VO}_{2} \right)-\left( 1.67* {VCO}_{2} \right)-(0.307 {PRO}_{oxidation})$ |
| **PRO oxidation (g·min^-1^):**  $\frac{EE* 0.15}{16.74 kJ}$ |
| **Energy expenditure (EE) (kJ·min^-1^):** $\left( 4.187 \right)* \left( 3.941* {VO}_{2} \right)+\left( 1.106* {VCO}_{2} \right)$ |

### *Sample Analysis*

To calculate RdT, fractional enrichments of intravenously administered [6,6-^2^H_2_]glucose were determined in the collected plasma samples by gas chromatography-mass spectrometry (GC-MS). Samples were deproteinised by adding 300 µL of ice-cold methanol to 50 µL of plasma and placed on ice for 30 min. Following centrifugation for 12 min at 12000 *g* at 4ºC, a volume of 300 µL supernatant was transferred to a glass screw neck vial and air-dried. Anhydride:pyridine (2:1) was added to the dry samples, vortex mixed, and left at room temperature for 30 min to form a penta-acetate glucose derivative. The samples were then analysed by GC-MS (6890N GC coupled with a 5975B MSD; Agilent Technologies) and in duplicates using electron impact ionisation and selected ion monitoring for measurement of isotope ratios. 1 µL of the sample was injected in splitless mode (injector temp. 280°C). Peaks were resolved using an HP5-MS 30 m × 0.25 mm ID × 0.25 μm capillary column (Agilent). Helium was used as carrier gas at 1.2 ml‧min^-1^ constant flow rate. The temperature ramp was set from 80 – 260°C at 15°C‧min^-1^. Selected ion recording conditions were used to monitor fragments m/z 200, 202 and 205 for glucose.

**Results**

**TABLE S1** Nutritional Composition of Experimental Drinks

|  | OMNI | VEG |
| --- | --- | --- |
| Energy, kJ | 1995 | 1998 |
| Energy, kcal | 476 | 479 |
| Carbohydrate, g | 74 | 74 |
| Protein, g | 18 | 18 |
| Fat, g | 12 | 12 |
| Fibre, g | 0 | 10 |

*The OMNI drink contains 250 mL full-fat milk, 50 g dextrose, 18.7 g dried skim milk and 9 g full fat dried milk The VEG drink contains 70 g dextrose, 40 g mycoprotein and 7 g olive oil.*

**TABLE S2** Macronutrient breakdown of dietary intakes at baseline and during the dietary intervention.

|  | OMNI | | | VEG | | |
| --- | --- | --- | --- | --- | --- | --- |
|  | Baseline | Intervention | %change | Baseline | Intervention | %change |
| Energy, kcal | 2022 | 2735 | 46 | 2258 | 2930 | 36 |
| Energy, kJ | 8460 | 11443 | 46 | 9447 | 12297 | 36 |
| Protein, g | 92.6 | 205.1 | 137 | 97.8 | 219.8 | 135 |
| *% Energy* | 18.5 | 30.0 | 72 | 17.8 | 30.0 | 74 |
| Carbohydrate, g | 204.7 | 239.3 | 21 | 229.0 | 256.4 | 17 |
| *% Energy* | 41.5 | 35.0 | -13 | 40.8 | 35.0 | -14 |
| Fibre, g | 24.4 | 42.5 | 74 | 26.0 | 65.2 | 160 |
| Fat, g | 89.1 | 106.4 | 35 | 98.2 | 114.0 | 33 |
| *% Energy* | 39.1 | 35.0 | -9 | 37.8 | 35.0 | -4 |

**TABLE S3** Sample one-day Meal Plan

| OMNI |  | VEG |  |
| --- | --- | --- | --- |
|  | Quantity |  | Quantity |
| **Breakfast** |  | **Breakfast** |  |
| Greek plain yoghurt | 200 | Alpro plain yoghurt | 300 |
| High-protein granola | 40 | High-protein granola | 40 |
| Peanut butter | 30 | Peanut butter | 30 |
| Blueberries | 30 | Blueberries | 30 |
|  |  |  |  |
| **Snack 1 (Protein shake)** |  | **Snack 1 (Protein shake)** |  |
| Whey protein | 45 | Pea protein | 50 |
| Semi-skimmed milk | 300 | High-protein soya milk | 300 |
|  |  |  |  |
| **Lunch (Omelette)** |  | **Lunch (‘Chicken’ Bagel)** |  |
| Eggs | 3 | Protein thin bagels | 120 |
| Goats cheese | 50 | Quorn ‘chicken’ slices | 100 |
| Spinach | 30 | Vegan mayonnaise | 15 |
| Tomatoes | 50 | Rocket | 45 |
|  |  | Cucumber | 20 |
|  |  |  |  |
| **Snack 2** |  | **Snack 2** |  |
| Rye ryvita | 60 | Vegan protein bar | 1 |
| Cottage cheese | 150 |  |  |
|  |  |  |  |
| **Dinner** |  | **Dinner** |  |
| Baked salmon | 150 | Quorn ‘chicken’ fillets | 250 |
| Baked sweet potato | 300 | Baked sweet potato | 200 |
| Baked asparagus | 75 | Baked asparagus | 75 |

*Sample plan contains 2385 kcals, with 180g carbohydrate, 205g protein and 90g fat. The OMNI plan contains 29.8g fibre and the VEG plan contains 63.1g fibre.*

**TABLE S4** Insulin sensitivity metrics obtained from the HEC.

|  | OMNI | | VEG | |  |  |  |
| --- | --- | --- | --- | --- | --- | --- | --- |
|  | Pre | Post | Pre | Post | *Group* | *Intervention* | *Group x Intervention* |
|  |  |  |  |  |  |  |  |
| EGP (mg٠kg^-1^٠min^-1^) | 2.3*±*0.2 | 2.2*±*0.2 | 2.6*±*0.1 | 2.5*±*0.2 | 0.200 | 0.054 | 0.855 |
| HIR (mg٠kg^-1^٠min^-1^٠mU^-1^٠L^-1^) | 44*±*12 | 45*±*19 | 71*±*44 | 68*±*36 | 0.060 | 0.899 | 0.782 |
| HIS (%) | 62*±*24 | 60*±*19 | 55*±*20 | 64*±*18 | 0.694 | 0.666 | 0.220 |
| HIS/ΔI (AU) | 1.0±0.5 | 1.1±0.3 | 1.0±0.3 | 1.4±0.5 | 0.334 | 0.049* | 0.224 |
| RdT (mg٠kg^-1^٠min^-1^) | 6.4*±*1.1 | 7.2*±*1.8 | 5.5*±*2.6 | 6.1*±*2.6 | 0.330 | 0.044* | 0.670 |
| PISI (mg٠kg^-1^٠min^-1^٠mU^-1^٠L^-1^) | 2.9*±*0.6 | 3.7*±*1.4 | 2.3*±*1.1 | 3.1*±*1.6 | 0.304 | 0.007* | 0.984 |
| PIS (fold increase in basal RdT) | 2.9*±*0.7 | 3.1*±*1.6 | 2.1*±*0.9 | 2.5*±*0.9 | 0.151 | 0.334 | 0.766 |
| GDR (mg٠kg^-1^٠min^-1^) | 5.7*±*0.5 | 6.2*±*0.6 | 5.4*±*0.7 | 5.7*±*0.6 | 0.723 | 0.270 | 0.894 |
| GDR/I (mg٠kg^-1^٠min^-1^٠mU^-1^٠L^-1^) | 2.8*±*0.6 | 3.6*±*1.5 | 2.4±1.2 | 3.3*±*1.5 | 0.580 | 0.014* | 0.894 |

*EGP= endogenous glucose production, HIR= hepatic insulin resistance (EGP*fasting serum insulin), HIS= hepatic insulin sensitivity (percentage suppression of EGP during Step 1 of HEGC), HIS/I= hepatic insulin sensitivity divided by change in serum insulin from basal to steady state of Step 1), RdT= rate of disappearance of glucose during Step 2 (high-dose insulin infusion), PISI= peripheral insulin sensitivity index (RdT ÷ mean insulin concentration during steady state of Step 2), PIS= peripheral insulin sensitivity, GDR= glucose disposal rate during Step 2, GDR/I = glucose disposal rate ÷ mean insulin concentration during steady state of Step 2. All data analysed by two-way ANOVA. Data presented as mean ± standard deviation.*

**TABLE S5** Pre and Post Physical Activity Data

|  | OMNI | | | | VEG | | | |  |
| --- | --- | --- | --- | --- | --- | --- | --- | --- | --- |
|  | Pre | | Post | | Pre | | Post | | *P*-value |
| Steps | 7620 | 3073 | 7465 | 2017 | 6662 | 3318 | 6401 | 3404 | 0.635 |
| Sleep | 6.2 | 0.7 | 6.4 | 0.8 | 6.2 | 1.2 | 6.6 | 1.0 | 0.308 |
| Sedentary | 13.0 | 1.2 | 12.8 | 1.1 | 13.5 | 1.2 | 13.6 | 1.1 | 0.793 |
| % Sedentary | 73.0 | 6.8 | 72.6 | 6.0 | 76.0 | 7.3 | 78.2 | 5.0 | 0.328 |
| Light | 2.9 | 0.8 | 3.1 | 0.8 | 2.4 | 0.8 | 2.0 | 0.9 | 0.430 |
| % Light | 16.2 | 4.9 | 17.6 | 4.8 | 13.5 | 4.1 | 11.4 | 5.5 | 0.540 |
| Moderate | 1.7 | 0.9 | 1.7 | 0.8 | 1.7 | 0.9 | 1.8 | 1.2 | 0.622 |
| % Moderate | 9.6 | 4.9 | 9.8 | 4.4 | 9.3 | 5.0 | 10.2 | 6.5 | 0.670 |
| Vigorous | 0.0 | 0.0 | 0.0 | 0.0 | 0.0 | 0.0 | 0.0 | 0.0 | 0.151 |
| % Vigorous | 0.1 | 0.1 | 0.1 | 0.1 | 0.0 | 0.0 | 0.1 | 0.1 | 0.164 |

*Mean and standard deviation shown.*

*All shown in hours per day.*

*Percentage calculations are calculated as a percentage of waking hours.*

*All data analysed by two-way ANOVA*


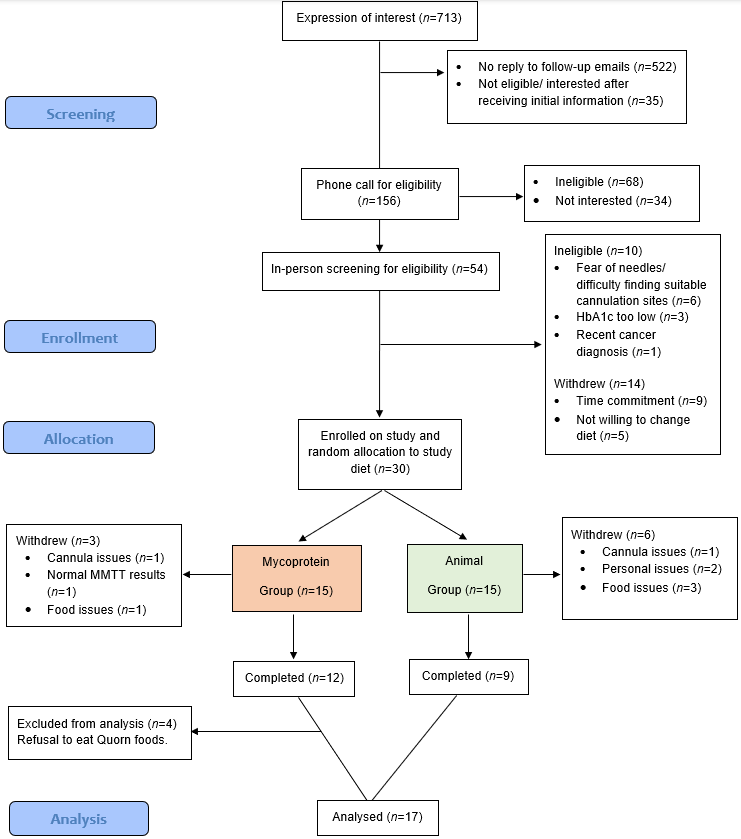


**FIGURE S1** CONSORT diagram

**FIGURE S2** Total dietary fibre intake per day in the OMNI and VEG groups. *denotes statistical significance (*P*<0.05) whereby the VEG group had a greater mean daily dietary fibre intake. Two-way ANOVA to assess change from habitual fibre intake to fibre intake during the intervention also reveals a significant effect of the intervention (*P*<0.0001) and also an interaction effect (intervention x dietary group *P*<0.0001).


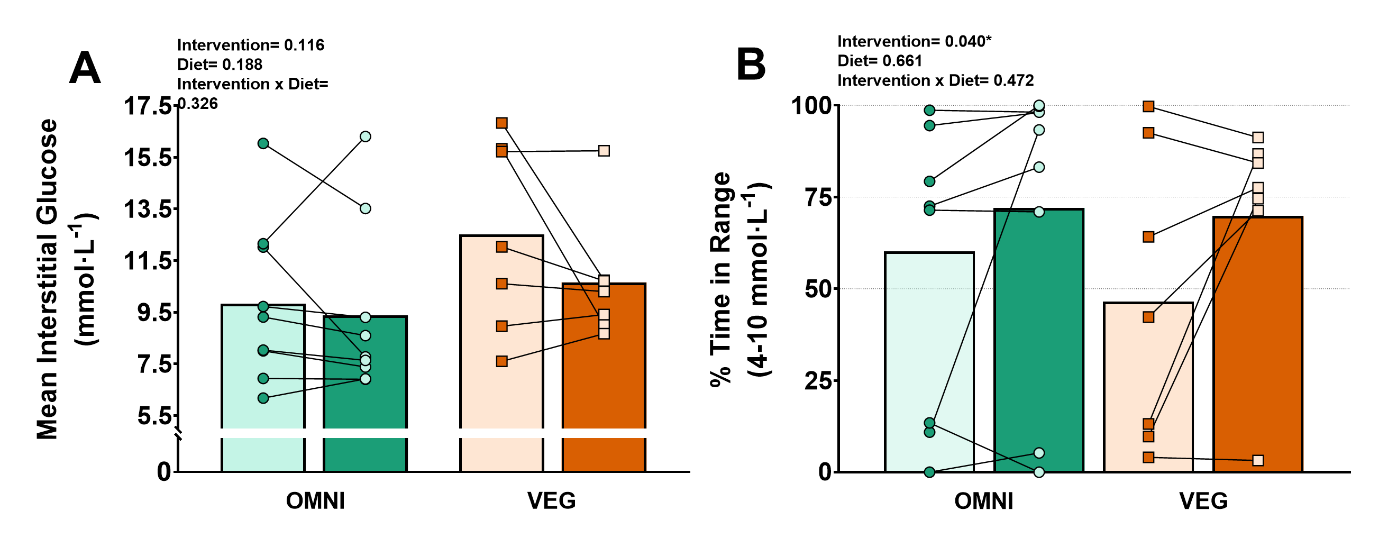


**FIGURE S3** Continuous glucose monitoring data. Change in mean interstitial glucose concentrations (A) and change in percentage time in range (between 4 and 10 mmol٠L^-1^) (B) in OMNI (circles) and VEG (square) groups from pre- to post-intervention. Lighter shades refer to pre-intervention and darker shades refer to post-intervention. Two-way ANOVA was used to analyse change in graphs A and B. *P*-values are shown on graphs.


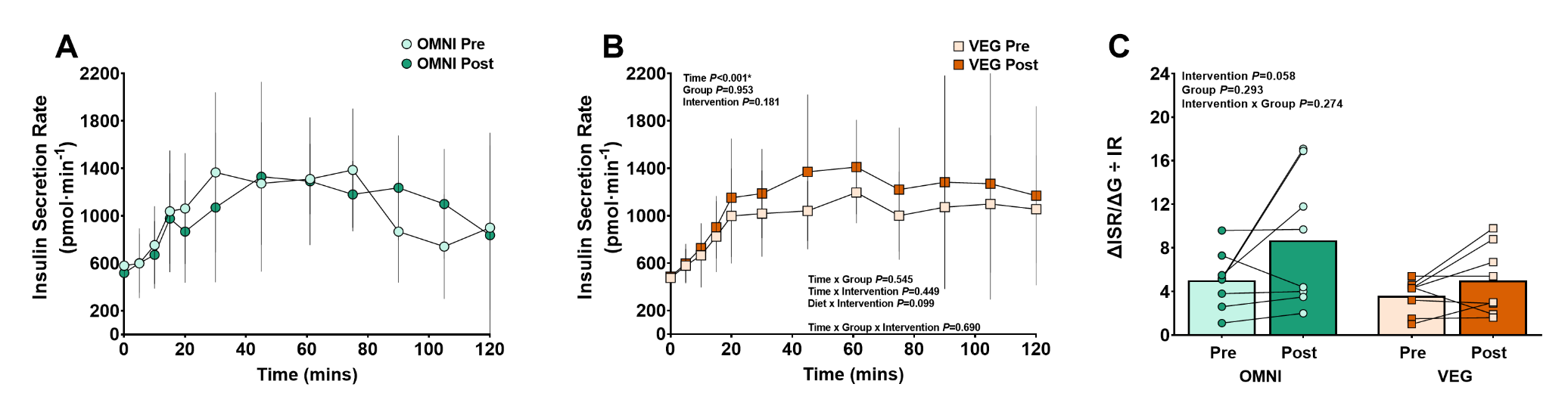
 **FIGURE S4** Insulin secretion rates during the mixed-meal tolerance test in the OMNI group (circles) (A) and VEG group (squares) (B). β-cell function is shown in C. The lighter shades depict pre-intervention secretion rates and the darker shades of colour depict post-intervention secretion rates. Error bars depict SD. *P*-values displayed represent results from three-way ANOVA.

**FIGURE S5** Model achieved of the hyperinsulinaemic-euglycaemic clamp. Constant rate of insulin infusion is represented by the dashed line and the variable infusion of glucose is represented by the line with circles for OMNI (A) and squares for VEG (B). Low-dose (30 mU) insulin infusion (step 1) ends after 150 minutes, and high-dose (80 mU) insulin infusion (step 2) begins. Shaded areas represent steady state. Elevations in the insulin infusion rate at the start of step 1 and step 2 reflect the priming dose administered. Mean blood glucose concentrations (consolidated data; OMNI and VEG, pre and post) during the steady state (120-150 min and 270-300 min) were 4.99 ± 0.11 and 5.05 ± 0.06 mmol٠L^-1^, which were equivalent pre- and post-intervention, and between groups (C and D). The lighter shade represents pre-intervention and the darker shade represents post-intervention. Dashed line at 5 mmol٠L^-1^ shown to depict target blood glucose concentration. Error bars depict SD. Mean insulin concentrations (consolidated data; OMNI and VEG, pre and post) are displayed in Figure C and D to demonstrate the increase in serum insulin concentrations with the increase in exogenous insulin infusion. Individual integrals were calculated between basal glucose concentration (*t_0_*) and 5 mmol٠L^-1^ (*t_n_*). Mean ± SD integrals pre- and post-intervention in OMNI were 424 ± 393 and 357 ± 312 mmol‧L^-1^‧*n* min^-1^ and 665 ± 214 and 479 ± 306 mmol‧L^-1^‧*n* min^-1^ in VEG, which was significantly lower post-intervention in both groups (intervention effect; *P=*0*.*017).

**FIGURE S6** Serum insulin concentrations (mU٠L^-1^) during the hyperinsulinaemic-euglycaemic clamp (A). Serum insulin concentrations (dashed line) and serum C-peptide concentrations (continuous line) in pmol٠L^-1^ during the HEC (B). Metabolic clearance rate (MCR) during the HEGC (C). Serum insulin concentrations increase from basal to step 1 and again to step 2 (insulin effect; *P<*0*.*001). Serum insulin concentrations post-intervention were significantly lower during the high-dose (step 2) insulin infusion (insulin x intervention effect; *P=*0*.*004). Serum C-peptide concentrations decreased significantly from basal to step 2 in both groups, both pre- and post-intervention (B). The intervention did not alter C-peptide concentrations in OMNI but caused a trend towards a decrease in VEG (*P=*0*.*076). MCR was calculated based on ratio of insulin infusion rate to change in insulin and C-peptide concentrations (C) and decreased during exogenous insulin infusion in both groups. After the intervention VEG trended towards an increased MCR at basal, step 1 and step 2 (group x intervention effect; *P*=0.052).

Data analysed using three-way ANOVA to compare concentrations between groups, intervention, and insulin dose. *P*-values refer to results from the ANOVA of insulin concentrations (graph A), C-peptide concentrations (graph B) and metabolic clearance rate (graph C). Error bars depicts SD. *represents statistical significance *P*<0.05. Serum C-peptide concentrations during the HEC are shown in Figure B with serum insulin concentrations (in pmol٠L^-1^) to demonstrate the inverse relationship between increased circulating serum insulin concentrations during exogenous insulin infusion, and simultaneous decreased endogenous C-peptide secretion.

**FIGURE S7** Carbohydrate oxidation (A) and storage (B) during the HEC. OMNI group data shown on the left using circles for individual data and VEG group data shown on the right using squares for individual data. Data analysed using three-way ANOVA to determine the main and interaction effects of insulin dose (insulin), OMNI or VEG dietary group (group) and intervention (pre and post). *P*-values are displayed on each graph. *represents statistical significance *P*<0.05.

1. Brouns, F., et al., *Glycaemic index methodology.* Nutrition research reviews, 2005. **18**(1): p. 145-171.

2. DeFronzo, R.A., J.D. Tobin, and R. Andres, *Glucose clamp technique: a method for quantifying insulin secretion and resistance.* American Journal of Physiology-Endocrinology And Metabolism, 1979. **237**(3): p. E214.

3. Frayn, K.N., *Calculation of substrate oxidation rates in vivo from gaseous exchange.* Journal of applied physiology, 1983. **55**(2): p. 628-634.

4. Steele, R., et al., *Measurement of size and turnover rate of body glucose pool by the isotope dilution method.* American Journal of Physiology-Legacy Content, 1956. **187**(1): p. 15-24.

5. Eaton, R.P., et al., *Prehepatic insulin production in man: kinetic analysis using peripheral connecting peptide behavior.* The Journal of Clinical Endocrinology & Metabolism, 1980. **51**(3): p. 520-528.

6. Cauter, E.V., et al., *Estimation of insulin secretion rates from C-peptide levels: comparison of individual and standard kinetic parameters for C-peptide clearance.* Diabetes, 1992. **41**(3): p. 368-377.

7. Gastaldelli, A., M. Abdul Ghani, and R.A. DeFronzo, *Adaptation of insulin clearance to metabolic demand is a key determinant of glucose tolerance.* Diabetes, 2021. **70**(2): p. 377-385.
